# Contraceptive use before first pregnancy by women in India (2005–2006): determinants and differentials

**DOI:** 10.1186/s12889-015-2652-y

**Published:** 2015-12-29

**Authors:** Anjali Pandey, K. K. Singh

**Affiliations:** Department of Statistics, Faculty of Science, Banaras Hindu University, 221005 Varanasi, India

**Keywords:** Contraception, Contraceptive use before first pregnancy, Logistic regression, Odds ratio

## Abstract

**Background:**

There exist ample of research literature investigating the various facet of contraceptive use behaviors in India but the use of contraception by married Indian women, prior to having their first pregnancy has been neglected so far. This study attempts to identify the socio demographic determinants and differentials of contraceptive use or non use by a woman in India, before she proceeds to have her first child. The analysis was done using data from the third National Family Health Survey (2005–2006), India.

**Methods:**

This study utilized information from 54,918 women who ever have been married and whose current age at the time of NFHS-3 survey was 15–34 years. To identify the crucial socio-demographic determinants governing this pioneering behavior, logistic regression technique has been used. Hosmer Lemeshow test and ROC curve analysis was also performed in order to check the fitting of logistic regression model to the data under consideration.

**Results:**

Of all the considered explanatory variables religion, caste, education, current age, age at marriage, media exposure and zonal classifications were found to be significantly affecting the study behavior. Place of residence i.e. urban - rural locality came to be insignificant in multivariable logistic regression.

**Conclusions:**

In the light of sufficient evidences confirming the presence of early marriages and child bearing practices in India, conjunct efforts are required to address the socio demographic differentials in contraceptive use by the young married women prior to their first pregnancy. Encouraging women to opt for higher education, ensuring marriages only after legal minimum age at marriage and promoting the family planning programs via print and electronic media may address the existing socio economic barriers. Also, the family planning programs should be oriented to take care of the geographical variations in the study behavior.

## Background

As per National Family Health Survey (NFHS-3) India: 2005–2006 report [1], 45 percent of women aged 20–24 were married by the age 18 years, 12 percent of women aged 15–19 years are already mothers; 4 percent of women 15–19 years are pregnant with their first child and in total, 16 percent women 15–19 years have begun childbearing. These figures clearly depict the existence of early marriages and early childbearing practices among Indian women which is a menace for the women and child welfare programs. The above cited report [1] renders the fact that in last three years preceding the NFHS-3 survey, there were 90 births per 1000 women aged 15–19 and 209 births per 1000 women aged 20–24 and this was highest of any age group. According to the same profile [1] these age-specific fertility rates indicate that, in India, a woman will have 1.5 children on an average by the time she has crossed her youth. Although fertility in urban areas is lower than that in rural areas, the urban age-specific fertility rates show that even in urban areas women have, on average, more than one child before they reach 25 years of age. Further, fertility during the youth years accounts for more than half (56 percent) of total fertility. Early age pregnancies are more likely to end in delayed or obstructed labor; ruptures in the birth canal; and associated death of mother, infant, or both. These risks are greater if prenatal care is inadequate [2]. Low birth weight, prematurity, birth injuries, stillbirth, and infant mortality are more likely to be associated with adolescent pregnancies [3]. Also due to lack of appropriate family life education young sexually active women are not only vulnerable to consider late, unsafe abortions as an alternative to carrying a pregnancy to term but also are subject to greater risks of infection with sexually transmitted diseases (including HIV/AIDS) compared to their older counter parts [4]. The fifth millennium development goal that aims to improve maternal health by 2015, especially the target 5B, also recognized the major concern of adolescent births, births to younger mothers and contraceptive prevalence. Health risks associated with early childbearing are not only factors which affect the quality of women’s life in any society but to some extent it also determines their role in the society as untimely pregnancy can force young women to discontinue their education, reducing their employment options later in life [5]. This also limits the intellectual development and economic utility of women. Consequently, for such women the crucial time of youth is spent in child bearing activities. Thus, delaying the first pregnancy, particularly in case of early marriages is desired for the health of mother as well as the child because one of the main conducing elements responsible for the morbidity and mortality among women of reproductive age groups are the complications associated with pregnancy and child birth, particularly in developing and under developed countries. Contraception, apart from being a proximate determinant of fertility, is one of the most important predictors of fertility transition. However, the use and choice of the contraceptive method is influenced by a number of interdependent socio - demographic variables and this calls for a multidimensional approach to probe the contraceptive use pattern. Also, the length of the interval between marriage and first live birth has always been considered as one of the most important factors determining the rate of the population growth. The longer the interval between marriage and first birth, slower is the rate of population growth, keeping the age at marriage fixed [6]. Thus lengthening the first birth interval may be considered as one of the important steps for regularizing the unregulated fertility. Apart from the above cited facts any attempt made towards postponement of the first pregnancy (consequently the first birth), indicates the existence of a planned vision towards family formation, which indeed is one of the prime objectives of almost all the family welfare programs.

This study intends to investigate the levels and trends of contraceptive use before first pregnancy by Indian women aged 15–34 years (who ever have been married) and identify socio demographic determinants governing the existing differentials in this behavior. In order to achieve the above mentioned intent logistic regression technique has been used which helps to observe how different explanatory variables affect the use of contraception before first pregnancy while controlling the other ones.

## Methods

### Data used

The present study is based on the NFHS-3 data, conducted in 2005–2006. The National Family Health Surveys are conducted by Ministry of Health and Family Welfare (India), International Institute for Population Sciences being the nodal agency. NFHS-3 provides data as well as estimates for fertility, mortality, family planning practices, maternal and child health, reproductive health, HIV/AIDS and awareness, nutritional status, utilization and quality of health and family planning services across 29 states/union territories and India as a whole. In NFHS-3, information from 124,385 women aged 15–49 years with a response rate of 94.5 percent was gathered. The information regarding contraception was furnished at various levels of details by asking more than thirty five questions from the respondents. This study utilized information from 54,918 women who have ever been married and whose current age at the time of survey was 15–34 years.

Further, in order to make a comparison among the percentages of women using contraception to prorogue their first pregnancy from NFHS-2 to NFHS-3, NFHS-2 data was also utilized. NFHS-2 data was also collected by Ministry of Health and Family Welfare (India) in year 1998–99. From NFHS-2 data we had 57,003 and 90,300 respondents in 15–34 and 15–49 years age groups respectively.

### Dependent variable, explanatory variables

**Fig. 1:**
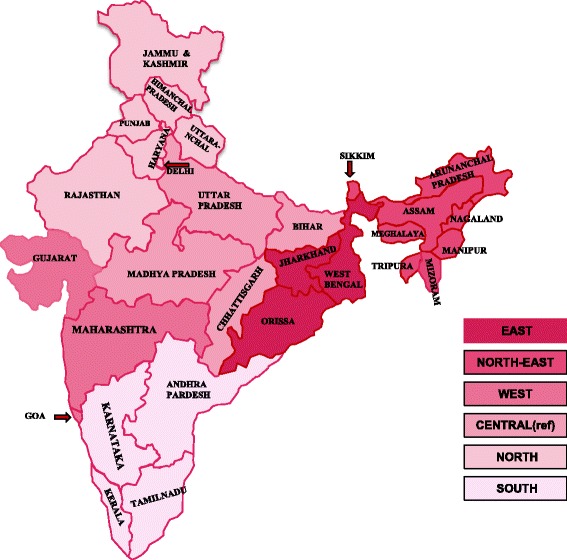
Classification of states/union territories considered in NFHS-3 under different zones

We concentrated only on one dependent variable defined for our study population which assigns a value 1 to all the women who opted for any contraceptive method to delay their first pregnancy and 0 to all those who either never used any contraceptive method or used it after having their first birth. In this study we have focused exclusively on the use of contraception prior to first pregnancy and not on the type of method used because of unavailability of information about this aspect. The explanatory variables used in the study are place of residence, religion, caste, educational qualification, current age, age at marriage, media exposure and Indian states/union territories clustered in zones. These variables seem to be affecting the use and non-use of contraception before first pregnancy and therefore, were investigated using logistic regression technique. Both crude as well as adjusted odds ratios were rendered in order to have better understanding of the extent to which each of the considered variables affect the phenomenon when we control the other variables under consideration. The place of residence represents the locality in which the respondent resides. Religion consists of three religious groups namely Hindu, Muslim and others. The others group comprised of Christians, Sikhs, Buddhist/Neo-Buddhist, Jain, Jewish, Parsi/Zoroastrian Donyi polo and others. The caste variable has been classed in SC/ST, OBC and others. The educational qualification was divided in to four categories - no or nominal education, primary education, secondary education and higher secondary education and above. The current age groups considered in our study are 15–19, 20–24, 25–29, and 30–34 years. The ages at marriage of the respondents were stratified in three groups as ≤16, 17–20 and ≥21 years. Further, media exposure is a composite variable educed from three questions: Do you read a newspaper or magazine almost every day, at least once a week, less than once a week or not at all?, Do you listen to the radio almost every day, at least once a week, less than once a week or not at all?, Do you watch television almost every day, at least once a week, less than once a week or not at all?. The final variable media exposure was recoded in to following three categories (1) not at all, if the answer to all the above three questions were “not at all”, (2) weekly or less than weekly exposure, at least any one of the answers being “at least once a week/less than once a week” and response to none of the three questions being “everyday”, (3) daily exposure, if the response to any of the above three questions is “every-day”. Twenty nine Indian states/union territories were covered for data collection in NFHS-3. These states/union territories were reorganized in to six zones namely north, central, east, north-east, west and south. This zonal classification has been provided in Table 1 and Fig. 1. The statistical analysis has been performed using the software package SPSS version 19.

**Table 1:** Classification of states/union territories under different zones

Zone	States/union territories included
North	Jammu & Kashmir, Himanchal Pradesh, Punjab, Uttaranchal, Haryana, Delhi, Rajasthan.
Central	Uttar Pradesh, Bihar, Chhattisgarh, Madhya Pradesh
East	Orissa, West Bengal, Jharkhand.
North-east	Sikkim, Arunachal Pradesh, Nagaland, Manipur, Mizoram, Tripura, Meghalaya, Assam.
West	Gujarat, Maharashtra, Goa.
South	Andhra Pradesh, Karnataka, Kerala, Tamil Nadu.

### Statistical methods

To identify the crucial socio-demographic determinants governing the pioneering study behavior, logistic regression technique has been used. The logistic regression technique is one of the most popular techniques used to assess the effects of multiple explanatory variables (continuous or categorical) on a dichotomous dependent variable i.e. the dependent variable assumes only two values (1 = success, 0 = failure). Hosmer Lemeshow statistics and Nagelkerke R square have been used to check how well the logistic regression model fits our data. Also, the ROC curve has been plotted to check the extent to which the logistic model correctly classifies the women under consideration as the users or non-users of contraceptive prior to the first pregnancy.

## Results and discussions

### Descriptive statistics of the respondents

**Fig. 2:**
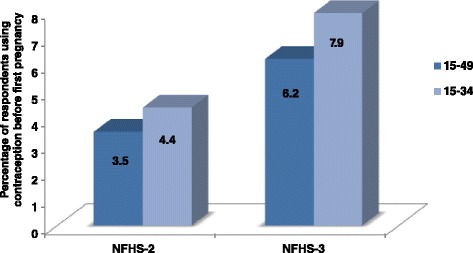
The difference in percent use of contraception before first pregnancy between NFHS-2 and NFHS-3 surveys

On investigating the NFHS-2 data for the study behavior under consideration it was observed that the percentage use of contraception to prorogue the first pregnancy has increased by 3.5 percentage points from NFHS-2 survey to NFHS-3 survey (Fig. 2) and reached 7.9 percent from 4.4 percent for the women who ever have been married and aged 15–34 years at the survey times. While for 15–49 year age consideration this increase is of 2.7 percentage points (from 3.5 percent for NFHS-2 to 6.2 percent for NFHS-3). This reflects increasing tendencies in efforts made towards contrived family formation between the two survey periods (1998–99 to 2005–06). This observed increase in the percentage points among the respondents, who used any contraceptive method before they were pregnant for the first time, lead to cogitation about the underlying socio demographic factors governing this characteristic.

Table 2 represents the percentage distribution of 15–34 years aged women who ever have been married and included in this study according to different socio-demographic attributes and contraceptive use prior to first pregnancy. As evident from the Table more than half of the respondents (58.4 percent) belong to rural areas in the data under consideration. At most three-fourth respondents belong to religion Hindu while 14.0 and 11.8 percent respondents belong to Muslim and others religious groups respectively. Having a caste wise composition of respondents it can be observed that 30.7 percent of them are from scheduled caste (SC) or scheduled tribes (ST), 33.7 percent from other backward classes (OBC) and 35.7 percent belong to others caste group. The educational composition of the women under study comprises that 35 percent respondents have no or nominal education, 15.2 percent have primary education whereas, majority, i.e., almost two fifth percent have secondary education and only 8.7 percent have education higher than secondary education. The current age structure of the women have been studied which consists 8.9 percent women belonging to age group 15–19 years while, 26.8, 33.2 and 31.1 percent of women belong to age group 20–24, 25–29 and 30–34 years respectively.

**Table 2:** Percentage distribution of respondents according to different socio demographic variables

Socio-demographic variables	Percentage distribution	Percentage women using Contraceptive before first birth
Place of residence		
Rural	58.4	6.5
Urban	41.6	9.8
Religion		
Hindu	74.2	8.3
Muslim	14.0	7.5
Others	11.8	5.8
Caste		
SC/ST	30.7	6.0
OBC	33.7	5.7
Others	35.7	11.6
Educational qualification		
No education	35.0	3.6
Primary	15.2	6.5
Secondary	41.1	9.1
Higher	8.7	22.0
Current age group		
15–19	8.9	13.1
20–24	26.8	9.9
25–29	33.2	7.3
30–34	31.1	5.4
Age at marriage		
< =16	39.0	5.5
17–20	41.1	7.7
> = 21	19.9	13.1
Media exposure		
Not at all	19.6	3.4
Weekly/less than weekly	25.1	6.8
Daily	55.2	10.0
Zones		
North	18.0	5.8
Central	23.2	6.3
East	12.5	16.0
North-east	15.5	10.5
West	12.4	8.6
South	18.3	3.8
Total	100	7.9
N	54,918	4336

From the tabulation of the variable age at marriage it was observed that 39 percent of the women were married at ages less than equal to sixteen years, 41.1 percent women got married between ages seventeen to twenty years and 19.9 percent women were married when their age was twenty years or more. Investigating for the degree of exposure it was noticed that 55.2, 25.1 and 19.6 percent of women are daily, weekly or less than weekly and not at all exposed to any kind of print or electronic media respectively. The distribution of women in six regional zones is as follows: north (18.0 percent), central (23.2 percent), east (12.5 percent), north-east (15.5 percent), west (12.4 percent) and south (18.3 percent).

It is worthwhile to mention that the percentage use of contraceptive prior to first pregnancy is higher (9.8 percent) for the women whose place of residence was urban as compared to women who were residing in rural areas (6.5 percent). This percentage is lower for the women belonging to Muslim religion (7.5 percent) and other religious groups (5.8 percent) as compared to the women who belong to Hindu families (8.3 percent). After analyzing the caste wise variation the percent of women trying to postpone the first pregnancy is highest for others caste group (11.6 percent) and almost equal for OBC (5.7 percent) and SC/ST (6.0 percent) category. According to expectations a positive relation is observed between use of contraception before first pregnancy and education of women. The percentage of women depicting this distinguished behavior increases with increase in educational level from 3.6 percent for no or nominal education group to 22 percent for higher educational group (higher than secondary). Further, with rise in the current age of women, the percentage of women using any contraceptive method to postpone their first pregnancy goes down from 13.1 percent for the age group 15–19 years to 5.4 percent for age group 30–34 years. While with increase in age at marriage the percentage of women portraying the study characteristic, increased by 7.6 percentage points from age at marriage less than equal to 16 years to age at marriage greater than equal to 21 years. It was observed that 5.5 percent women use any contraceptive prior to first pregnancy whose age at marriage being less than equal to 16 years and 13.1 percent women use any contraceptive before first pregnancy whose age at marriage is greater than equal to 21 years.

It could be notice that as the media exposure is increasing, the percentage use of contraception before the first pregnancy is also increasing. As for those who are not at all exposed to media this percentage is 3.4, for those with weekly or less than weekly exposure it is 6.8 percent while 10.1 percent of the respondents with a daily exposure to media went for using contraceptives before they have had any pregnancy. Geographical variations were marked as east zone has highest percentage of women (16.0 percent) depicting the study characteristic and north-east stands second to east with 10.5 percent. South zone has the least 3.8 percent women who used contraception before their first pregnancy. These percentages for North and central zones were 5.8 percent and 6.3 percent respectively.

### Logistic regression analysis

In order to verify whether the differences offered by the different layers of the various explanatory variables considered in our study are statistically significant or not and how the movement from one social or demographic strata to another affects the study behavior, we have applied logistic regression technique and obtained crude as well as adjusted odds ratios. In order to rule out the presence of multicollinearity, the matrix of correlations of estimates was generated and each of the correlation values was much smaller than 0.8. Also, each independent variable in this analysis had a standard error less than 2.0. Both these criterion leave us with no evidences of multicollinearity. The prediction accuracy for our model is 92.2 percent and the *p* value for Hosmer Lemeshow statistics is 0.839 (>.05) implying that our logistic model fits the data quite well. The value of Nagelkerke R square (pseudo R square) is 0.181 suggesting that our model is a strong improvement over a null model with no predictors.

One of the primary objectives of executing logistic regression is to bring forth an equation that can accurately classify the observations in to one of the two possible outcomes. The degree to which the predictions agree with the data may be shown graphically by either a receiver operating characteristic curve (ROC) or an overlay plot of sensitivity and specificity versus predicted probabilities [7–9].The ROC curve plots the sensitivity versus (1- specificity). The value of the area under curve for ROC curve ranges from 0.5 to 1.0 with larger values indicating a better t. Area under curve for our model is 0.776 with 95 percent confidence intervals (0.769, 0.783). Also, the area under the curve is significantly different from 0.5 as the *p*-value is much less than 0.001 which suggests that logistic regression classifies the two groups (i.e. users and non-users of any contraceptive method before first pregnancy) significantly better than by chance.

Table 3 exhibits the result of logistic regression which gives the crude as well as adjusted odds ratios with their confidence intervals for the study characteristic, i.e., use of contraception after marriage before first pregnancy. Except few categories, rest of the predictor variables were found to have significant influence on the contraceptive practices before first pregnancy in both univariate as well as multivariable logistic regression models.

**Table 3:** Logistic regression results of contraceptive use before first pregnancy by 15–34 years aged Indian women’s (who were ever married) socio demographic characteristics

Socio-demographic variables	Crude odds ratio			Adjusted odds ratio		
	Odds ratio	C. I. (95 %)		Odds ratio	C. I. (95 %)	
		Lower	Upper		Lower	Upper
Place of residence						
Rural	ref	-	-	ref	-	-
Urban	1.558**	1.464	1.658	0.983	0.913	1.058
Religion						
Hindu	ref	-	-	ref	-	-
Muslim	0.900*	0.822	0.987	0.840**	0.758	0.931
Others	0.681**	0.610	0.760	0.499**	0.440	0.566
Caste						
SC/ST	ref	-	-	ref	-	-
OBC	0.814**	0.736	0.889	0.993	0.900	1.096
Others	1.755**	1.604	1.920	1.330**	1.214	1.458
Educational qualification						
No education	ref	-	-	ref	-	-
Primary	1.885**	1.680	2.116	1.401**	1.240	1.583
Secondary	2.702**	2.472	2.952	1.899**	1.710	2.108
Higher	7.635**	6.892	8.459	5.705**	4.975	6.543
Current age group						
15–19	ref	-	-	ref	-	-
20–24	0.724**	0.656	0.799	0.579**	0.520	0.644
25–29	0.518**	0.468	0.572	0.332**	0.296	0.372
30–34	0.377**	0.339	0.419	0.229**	0.203	0.258
Age at marriage						
< =16	ref	-	-	ref	-	-
17–20	1.427**	1.322	1.541	1.196**	1.099	1.303
> = 21	2.597**	2.396	2.816	1.687**	1.563	1.941
Media exposure						
Not at all	ref	-	-	ref	-	-
Weekly/less than weekly	2.085**	1.843	2.359	1.709**	1.497	1.950
Daily	3.152**	2.821	3.521	2.034**	1.784	2.320
Zones						
North	0.986	0.885	1.099	0.816**	0.728	0.916
Central	ref	-	-	ref	-	-
East	4.383**	3.978	4.830	4.774**	4.296	5.306
North-east	1.864**	1.691	2.055	2.124**	1.904	2.370
West	1.493**	1.339	1.664	1.152*	1.027	1.292
South	0.624**	0.552	0.706	0.498**	0.438	0.566

* *p* value < .05, ** *p* value < .01

Having a glance at the crude odds ratio one concludes that odds for using any contraceptive method before first pregnancy is 1.55 times higher for a respondent belonging to urban area as compared to one from rural area and it is highly significant. But as soon as the procedure is controlled for all the other explanatory variables, the difference between rural and urban place of residence remains no more significant.

There are highly significant variations according to the religious affiliations in use of contraception by the women before first pregnancy. The odds ratios for Muslim women and women belonging to other religious groups are .840 and .499 respectively. This leads us to infer that compared to women belonging to Hindu religious affiliation, those having Muslim or other religious affiliations are less likely to use any contraceptive method before their first pregnancy controlling other variables under study. From the result it is evident that religious affiliation affects the study characteristics in certain manner which may be ascribed to some under lying religious believe and practices. This result is in consensus with findings of few similar studies [10, 11].

Further, considering the caste wise classification of the respondents the adjusted odds ratio increased significantly to 1.33 for the women belonging to others caste group taking the women who belong to SC/ST as reference group, i.e. a woman from others caste has 1.33 times higher odds to use any contraceptive method in order to delay her first pregnancy as compared to the women belonging to SC/ST caste. This ensuing variation is in agreement with previous studies investigating the contraceptive behavior [12, 13].

A women’s level of education elevates her chances of using contraceptive prior to her first pregnancy. As compared to a woman with no or nominal education, the one having primary education had 1.401 times higher odds and those with secondary education had 1.899 times higher odds of using contraceptive before they have had their first pregnancy. Immense hike in the odds ratio (5.705) has been evoked for the highest level of education. This means that the women with education more than secondary level has 5.7 times higher chances to use contraception prior to having any child than the women who have no or nominal education. This finding clearly is one of the high spots of this study and it has been found in previous studies as well [14–17] that women’s education is a strong determinant governing their contraceptive behavior. This is an indication that highly educated women are more liable to adopt any method to delay their first pregnancy. From this finding one may deduce, therefore, with certitude that educated women are more aware about the significance of planned family formation approach.

Considering the age composition, increase in current age is accompanied with declining odds ratios for adopting any mean to regulate the first pregnancy. Taking the women belonging to age group 15–19 years as reference; the odds for contraceptive use prior to first pregnancy for the women aged 20–24 years, 25–29 years and those aged 30–34 years are .579, .332 and .229 respectively. In line with the declining odds ratio we conclude that the younger generation of married women are more likely to regulate the commencement of their child bearing phenomenon as compared to their older counter parts. This outcome being in accord with a youth report of NFHS-3 [1] and previous studies investigating the contraceptive behavior [18] suggests appreciation of contrived movement in family building process by younger generations.

Increase in age at marriage is followed by highly significant increments in the odds of using contraceptive prior to first pregnancy. It was observed that the odds ratio goes up to 1.196 for the women marrying at ages ranging from seventeen to twenty years and ascends to 1.687 for the women whose age at marriage being twenty one year or more; women with age sixteen year or less being the reference category. Earlier studies probing the contraceptive behavior of women also supported the fact that increase in age at marriage removes the layers of vulnerability for poor fertility regulating behavior [19, 20]. This may be due to fact that as the age at marriage rises, the woman becomes more mature and is capable of communicating with her spouse and making decisions about her contraceptive behavior. Being in line with prior studies [21–27] this study also unraveled that exposure to media does influence the contraceptive practices. With increasing levels of exposure to media, there is rapid hike in the odds ratios. As compared to the respondents with no media exposure at all, those with weekly or less than weekly exposure to any kind of print or electronic media are 1.709 times more likely to use any contraceptive method to delay their first pregnancy. Further, the women with daily exposure to any kind of media (television, radio, and news paper) are approximately 2.034 times more likely to do so and the result is statistically significant. This finding leads us to infer that exposure to print and electronic mass media is substantial factor affecting positively the contraceptive use behavior of women aged 15–34 years prior to first pregnancy. Media exposure being a source of various government sponsored family planning messages, steered the women soliciting guidance.

The differentials offered by regional composition are remarkable. As compared with central zone we found that the women from north and south regions are less likely. The odds of adopting any contraceptive method to delay the first pregnancy for northern women are .816 and that for southern women .498, taking central zone as reference. The odds ratio for women belonging to western zone goes up to 1.152 pointing towards rise in chances of depicting the study characteristic than the women who live in central zone. The north-east and east zones stand far apart with odds ratio being more than 2 times and 4.7 times respectively to use contraceptive before first pregnancy. With all the other predominant variables being controlled, the zones are offering ineluctable variations. This evokes an obvious curiosity to further investigate the factors influencing the variation across the zones. The cultural variations across the zonal classification may be plausible reasons for the underlying spatial variation.

## Conclusions

This study canvassed the socio demographic factors determining the trends and differentials in the use of contraceptive methods before first pregnancy. By having a glance at Table 2 one can see that almost two- fifth of the women were married at the ages less than equal to 16 years and same proportion were married between 17 years to 20 years. With this large proportion of young women entering into sexual union and a very small percentage of them (5.5 and 7.7 % respectively) were adopting any method of contraception to postpone their first pregnancy. According to a study done by Economics and Statistics Administration, U.S. Bureau of the Census [2], About 15 million babies are born to adolescent mothers each year. About 8 in every 10 of these babies are born in the developing countries of Asia, Africa, and Latin America. And about 13 percent of all children born in developing countries are born to teenage mothers. These are high-risk births from the perspective of the health of both mother and child. They are also high-cost births when the associated negative effects on the quality of life and role of women in society are considered. Reproductive health is a particular concern in the case of early adolescent pregnancy and childbearing, i.e., where the mother is age 17 or younger rather than age 18 or 19”. Thus it can be strongly recommended to the policy planners that they should prioritize the need to focus on the factors affecting the use or non-use of family planning methods, by young vulnerable women before they start their family formation. After investigating the variables which are supposed to influence the considered study character at individual and household levels this study came upon with religion, caste, education, current age group, age at marriage, media exposure and states/union territories classified in zones as the significantly influencing factors. Though overall employment of any efforts to postpone the first pregnancy is very low (7.9 percent), the variations offered by religion suggests that Muslims and others religious groups should be given slightly high attention as compared to Hindus to use contraceptive before first pregnancy. Similar suggestion holds for the women belonging to SC/ST caste group being compared with the women who belong to the household of others caste category.

Highly significant increments in odds ratios with increase in educational level suggests that in order to combat the problems associated with early motherhood and to promote a planned parenthood approach, women should be encouraged to go for higher levels of education. Further, the hike in odds ratios accompanied with increasing age at marriage suggests that the policy planners should try to ensure that marriages take place only after legal minimum age of marriage by implementing the existing laws more effectively and spreading awareness among common citizens about the stringent penalization provisions for violating these laws. Also government should run interventions and programs targeting the intellectual and skill development of the young married women, and put more efforts to spread awareness about the health and personal hazards which early childbearing practices can pose to young women.

Media exposure has also emerged as one of the key variables having significant influence over the phenomenon, so our suggestion to the policy planners is to intensify the already existing levels of family planning program’s promotion through print and electronic media and to ensure that more and more women and couples should be exposed to such programs through print and electronic media. The zonal variation seems to exist due to underlying socio-cultural faiths and practices. More precise studies should be done to identify the factors offering this variability.

## Abbreviations

SC/ST: Scheduled castes/Scheduled tribes
OBC: Other backward classes
NFHS: National family health survey
